# ABCG2 Transporter: From Structure to Function—Current Insights and Open Questions

**DOI:** 10.3390/ijms26136119

**Published:** 2025-06-25

**Authors:** Laura Álvarez-Fernández, Alicia Millán-García, Gracia Merino, Esther Blanco-Paniagua

**Affiliations:** Department of Biomedical Sciences–Physiology, Faculty of Veterinary Medicine, Instituto de Desarrollo Ganadero y Sanidad Animal (INDEGSAL), University of León, Campus de Vegazana s/n, 24071 León, Spain; lalvf@unileon.es (L.Á.-F.); amilg@unileon.es (A.M.-G.); gmerp@unileon.es (G.M.)

**Keywords:** ABCG2, structure, transport, conformation

## Abstract

ABCG2 is a crucial ATP-binding cassette (ABC) transporter involved in multidrug resistance and essential physiological and pharmacological processes. In recent years, multiple ABCG2 structures have been resolved using cryo-electron microscopy (cryo-EM), providing significant insights into its conformational states during its transport cycle. However, even more than 25 years after its description, a high-resolution X-ray crystallographic structure is still unavailable, limiting the understanding of its dynamic transitions, as well as leaving aspects of the transport cycle unresolved and open to discussion. Given the complexity of ABCG2, a multidisciplinary approach is essential in order to fully elucidate its mechanism. This review compiles recent advances in ABCG2 structural biology, highlights unresolved controversies, and explores future directions to bridge the gap between structure and function. Moving forward, integrating multiple structural and functional approaches will be key to uncovering the intricate workings of this enigmatic transporter. In particular, detailed structural insights will be crucial to identifying new ABCG2 substrates and designing selective inhibitors, with important implications for therapeutic development.

## 1. Introduction

ABC transporters constitute one of the largest superfamilies of integral membrane proteins that mediate the transport of a wide variety of substrates across membranes using energy derived from ATP hydrolysis [1]. Their structure, membrane topology, and mechanism of action are highly and evolutionarily conserved throughout all living organisms, ranging from ancestral prokaryotes to advanced eukaryotic systems [2]. Whereas prokaryotes use both ABC importers and exporters, eukaryotic organisms, with rare exceptions, encode primarily ABC exporters [3].

Following the classification framework applied to animal ABC proteins [4], which is derived from phylogenetic analyses of the nucleotide-binding domain (NBD) amino acid sequences, as well as differences in domain orientation and overall protein size, they are categorized into eight subfamilies from ABCA to ABCI [5,6], encoded by 48 genes in humans [7]. Notably, while the ABCH subfamily is present in animals, it is absent in plants, where the ABCI subfamily functionally replaces it [8]. They can act as receptors, ion channels, and membrane transporters [9], or be involved in mRNA translation control, in the case of members of the subfamilies ABCE and ABCF [10].

The canonical structure of an ABC transporter consists of four functional units or domains, two highly conserved NBDs and two transmembrane domains (TMDs) arranged in different configurations. Particularly, in eukaryotes, ABC transporters can be constituted by a single peptide (full-transporter) or two polypeptides that undergo dimerization (half-transporter), which contain all four functional units [3,11,12,13]. Notwithstanding, ABC subfamilies present different domain organizations. While members of the ABCA to ABCD subfamilies have a forward TMD-NBD domain arrangement, their organization is in reverse order in members of the ABCG subfamily [12]. Alternatively, the soluble ABCE and ABCF subfamilies lack TMDs [12,14], containing only two NBDs, and the ABCI subfamily possesses merely a single domain, either an NBD, a TMD, or an alternative accessory domain [12].

The ABCG proteins comprise the largest known subfamily [15], to which the ABCG2 protein belongs. ABCG2, also known as breast cancer resistance protein (BCRP), is a half-transporter [9] that acts as an efflux pump by expelling out of the cells a plethora of structurally diverse compounds, not only drugs and xenobiotics but also endogenous and dietary compounds, accordingly affecting their bioavailability and their therapeutic efficacy in the case of drugs [16,17,18,19]. In the case of xenobiotics, its function is mainly protective, avoiding the accumulation of toxins in the body [20].

Although ABCG2 is expressed in the mitochondrial membrane, its expression has predominantly been described in the apical membrane of cells [21] of physiological, and pharmacology- and toxicology-relevant organs involved in the absorption, distribution, and excretion of its substrates [19]. These organs include the liver, the kidney, the gastrointestinal tract, the male and female reproductive systems [22], and the lactating mammary gland [23,24], being the only ABC transporter involved in the active milk secretion of its substrates [17,25,26,27]. It is also expressed in the vascular endothelium of the main physiological barriers, including the blood–brain, blood–testis, and fetoplacental barrier [28], and in stem-cell-enriched cell populations and progenitor cells of different tissues [29,30]. On the other hand, ABCG2 is overexpressed in tumor cells, as well as other ABC transporters such as P-glycoprotein (P-gp/ABCB1) and multidrug-resistance-associated protein 1 (MRP1/ABCC1) [31], whose ability to expel chemotherapeutic agents out of the cells confers failure in antitumor therapies, along with the widely known phenomenon of multidrug resistance (MDR) [32,33,34].

Based on the aforementioned localization and physiological functions, it is of the utmost importance to point out that changes in ABCG2 expression and activity can modify the pharmacokinetics and bioavailability of any of its substrates due to the existence of several polymorphisms [35,36], the coadministration of several drugs, the drug–food interactions, or interactions with other endogenous substrates [37]. Consequently, ABCG2 is on the US Food and Drug Administration and the European Medicines Agency lists of transporters to be checked for drug–drug interactions [38].

Accordingly, to develop therapeutic modulators of ABCG2 transport activity, it is crucial that we thoroughly describe the precise 3D structure of the transporter [9]. In recent decades, in vivo studies have yielded comprehensive knowledge regarding the localization, physiological roles, trafficking, substrate specificity, and single-nucleotide polymorphisms of ABCG2 [39,40,41]. However, after the first publication of its cDNA and protein sequence in 1998 [42] and the description of more than 20 ABCG2 cryo-EM structures at various resolutions and in diverse conformational states [43], a high-resolution X-ray crystallographic structure is still unavailable due to the challenges related to its overexpression and functional purification, hampering the understanding of its mechanism of transport.

Early attempts to elucidate the structural organization of ABCG2 relied on low-resolution electron microscopy and mechanistic prediction in silico models based on other ABCG transporters. Specifically, László et al. (2016) built the first homology model rooted in the structural data obtained from the X-ray diffraction of the sterol transporter ABCG5/8, which belongs to the same subfamily as ABCG2 [44]. Although the overall architecture of ABCG2 closely resembles the ABCG5/G8 fold, which is highly conserved across the ABCG family, key functional differences could not be predicted by in silico models. Notably, ABCG5/G8 displays a narrow substrate specificity, primarily restricted to sitosterol and cholesterol, whereas ABCG2 can transport a remarkably broad range of substrates [3]. To shed light on this functional divergence, Taylor et al. reported in 2017 the first high-resolution (3.8 Å) cryo-EM structure of human ABCG2 [38]. Since then, a series of complementary studies has improved our understanding of its conformational dynamics, substrate and inhibitor binding sites, and transport mechanism of ABCG2 through a combination of cryo-EM data, molecular docking, free binding energy calculations, molecular dynamics simulations, and genetic and functional assays [18,43,45].

Unfortunately, most of the mechanistic details regarding substrate selectivity, ligand recognition, and translocation remain elusive. The integration of diverse structural and functional methodologies will be crucial for fully elucidating the complex mechanisms underlying this enigmatic transporter. In this sense, this review gathers the latest insights into ABCG2 structural biology, addresses unresolved controversies, and describes potential approaches to link structure and function.

## 2. Molecular Structure of the Human ABCG2 Transporter

**Figure 1:**
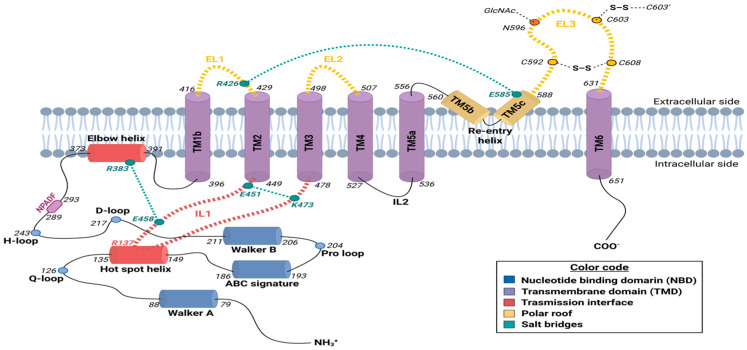
**Schematic representation of the structural topology of each monomer of the half-transporter ABCG2.** In the cytoplasmic side, the main conserved domains of the nucleotide-binding domain (NBD) are shown in blue. In the transmembrane region, the six transmembrane helices (TMs) are represented in purple, along with the elbow helix (red) and the re-entry helix (yellow). The transmission interface is highlighted in red, and structural elements involved in the formation of the polar roof are shown in yellow. The start and end positions of each conserved motif and helix within the peptide sequence are indicated. In the extracellular loop 3 (EL3), the cysteine residues (C592, C603, and C608) involved in disulfide bond formation are marked, as well as the N-glycosylation site (N596). Finally, residues involved in salt bridge formation are shown in green. Created in https://BioRender.com.

The human ABCG2 transporter is a protein encoded by a 66 kb gene, which is located on the long arm of chromosome 4 (4q22.1) and organized into 15 introns and 16 exons. This sequence encodes a 655-amino-acid, 72 kDa protein with a half-transporter conformation that requires homodimerization to be completely functional. Accordingly, the quaternary structure of ABCG2 comprises a 144 kDa homodimer formed by two identical polypeptide chains [38], each one with a N-terminal cytoplasmic NBD (residues 1 to 396), followed by the transmission interface—including the highly conserved triple helical bundle (THB) cluster—and the C-terminal TMD (residues 397 to 655), consisting of six transmembrane helices (TMs), with the re-entry helix at the outer membrane leaflet (Figure 1). This “reverse” configuration characteristic of the ABCG subfamily, with the domain arrangement N-NBD-TMD-C on the primary protein sequence, contrasts with other eukaryotic ABC transporters, as indicated in the previous section [9,46].

### 2.1. Nucleotide-Binding Domain (NBD)

Early high-resolution cryo-EM structural studies of ABCG2 failed to resolve the NBD with sufficient density, largely due to the high intrinsic flexibility of these domains, which is, at the same time, essential for ATP binding and the transport cycle. It was not until 2018 that a complete, de novo high-resolution structure of the ABCG2 NBD was successfully obtained [45]. Until then, structural features were mainly inferred from other ABC transporters, as the NBD sequence is highly conserved across the entire ABC superfamily. These predictions were subsequently validated by biochemical data, supporting the notion of a conserved general NBD structure among all ABC proteins [47].

Focusing on ABCG2, it is noteworthy that the two NBD domains form a symmetric homodimer. In the absence of ATP, the NBDs adopt an open conformation at their nucleotide-binding sites, with an approximate 35° angle, to facilitate nucleotide exchange. Upon ATP binding, the nucleotides become occluded, a feature shared with other ABC transporters. Interestingly, in ABCG2, the NBD dimer remains continuously connected despite the absence of the bound nucleotide [47,48]. This interaction is mediated through a conserved motif in all of ABCG subfamily (consensus sequence NPxDF—NPADF in ABCG2–; residues 289–293) located at the C-terminal end of the NBD (Figure 1), thereby providing structural stability during the dynamic rearrangements required throughout the transport cycle [45,49,50].

**Figure 2:**
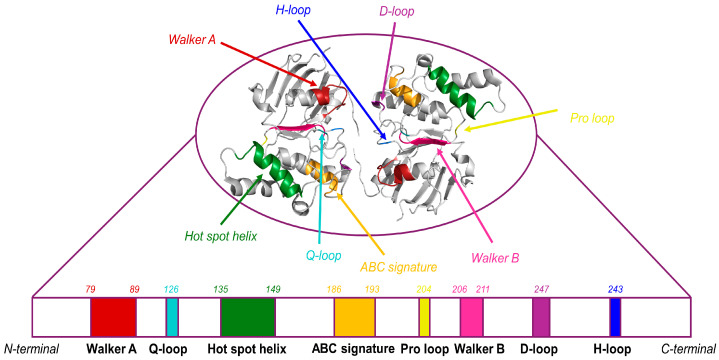
**Structural organization of the conserved NBD in ABC transporters.** The motifs involved in ATP binding and hydrolysis are represented in different colors, from the N-terminal to the C-terminal end: Walker A (red), from residue 79 to 88; Q-loop (light blue), residue 126; hot spot helix (green), from residue 135 to 149; ABC signature (orange) from residue 186 to 193; pro loop (yellow), residue 204; Walker B (pink) from residue 206 to 211; D-loop (purple) from residue 216 to 217; and H-loop (dark blue), residue 243. For visualization of the NBD, the PyMOL Graphics System v.3.1.5.1 was used (Schrödinger, LLC., New York, NY, USA). Based on Badiee et al. (2023) [55].

In the human ABCG2, the NBD structure contains highly conserved motifs shared among all of the ABC family which, in order of proximity to the N-terminal end, are known as the Walker A motif (also referred to as the phosphate-binding loop or P-loop; consensus sequence GxxGxGK(S/T), with x representing any amino acid; residues G79 to S88), Q-loop (residue Q126), hot spot helix (also known as E-helix; T135 to A149), ABC signature motif (also termed as C-loop; consensus sequence L/VSGGQ/E—VSGGERKR in ABCG2–; residues V186 to R193), pro-loop (residue P204), Walker B motif (consensus sequence hhhhDE, with h standing for any hydrophobic residue; residues I206 to E211), D-loop (residues L216 and D217), and histidine switch (also known as H-loop; residue H243) (Figure 2) [18,51,52,53]. The presence and functional relevance of the A-loop motif—also conserved among ABC transporters—remain controversial in the case of ABCG2 [9,54].

ATP binding occurs in the commonly known catalytic site of the protein, located between the Walker A motif—a glycine-rich sequence with a conserved lysine (K) residue able to form hydrogen bonds with the β- and γ-phosphates of the ATP molecule—and the ABC signature motif, which is a hallmark exclusive to ABC transporters [9,54]. Furthermore, the Walker B motif—which includes a conserved aspartate (D) and a glutamate (E) residue—along with the conserved glutamine (Q) from the Q-loop, coordinates the Mg^2+^ ion association with the ATP together with the water molecule needed for the ATP hydrolysis. The bound water molecule is polarized with the help of the Walker B, H-loop, D-loop, and Q-loop, favoring the nucleophilic attack on the γ-phosphate of ATP, thus resulting in ADP and free phosphate generation [52,56]. The Q- and D-loops are also responsible for NBD dimer formation [53].

Interestingly, the hot spot helix (Figure 1)—an essential component of the transmission interface, as will be described in the following section—is a groove-like structure located at the top of the NBD dimer. This helix provides the structural docking platform for the insertion of intracellular loop 1 (IL1), thereby facilitating the integration of the coupled movements between the cytosolic and transmembrane domains [57]. The hot spot helix includes some relevant highly conserved residues essential for protein folding and function, including the positively charged R137, and the negatively charged E138, Q141, and F142, all of them facing the elbow helix and playing a key role on ABCG2 the stability of NBDs. Q141 directly interacts with IL1, likely contributing to protein folding stability, thereby providing a structural basis for the impact of the gout-associated Q141K polymorphism [58,59].

Other residues of interest within the NBD include Q181 and F182, facilitating contacts between the TM1 of one ABCG2 monomer and the TM5 of the other, thus helping stabilize the TMD domain closure; and the arginine residue R184, providing a direct contact with the adenine nucleotide of the ATP molecule bound to the opposite NBD [20].

Due to spatial and structural constraints, not all of the structural elements within a single NBD can be arranged around an ATP molecule. Thus, the two ATP molecules bind simultaneously at the interface of the NBD dimer, using the missing structural elements from the opposing NBD in a complementary manner. Specifically, ATP interacts with the Walker A motif, Q-loop, Walker B motif, and H-loop of one NBD, and with the ABC signature motif and D-loop of the opposing NBD [9].

### 2.2. Transmembrane Domain (TMD)

Within the ABC transporter superfamily, the TMD exhibits significantly lower sequence conservation compared to NBD. The divergence is attributed to the role of TMDs in determining the substrate specificity, being evolutionary adaptations necessary for accommodating the distinct functional requirements of individual transporters [3,60].

**Figure 3:**
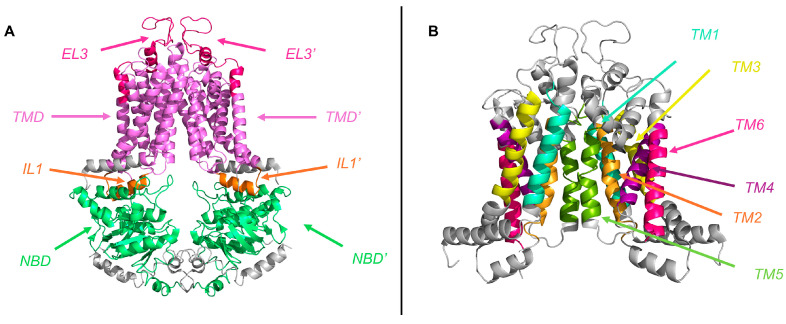
(**A**) **Structural representation of the human ABCG2 transporter.** Domains are represented in different colors: nucleotide-binding domain (NBD-NBD′) (light green) and transmembrane domain (TMD-TMD′) (light purple), and, furthermore, intracellular loop 1 (IL1-IL1′) (orange) and extracellular loop 3 (EL-EL3′) (pink). (**B**) **Structural organization of the TMD in ABCG2 transporter**. Six α-helical TM are represented in different colors, from the N-terminal to the C-terminal end: TM1 or TM1b, from residue 396 to 416 (light blue); TM2, from residue 429 to 449 (orange); TM3, from residue 478 to 498 (yellow); TM4, from residue 507 to 527 (purple); TM5 or TM5a, from residue 536 to 556 (green); and TM6, from residue 631 to 651. For visualization of both molecular structures, the PyMOL Graphics System v.3.1.5.1 was used (Schrödinger, LLC., New York, NY, USA).

The TMD of ABCG2 is composed of six α-helical TM (TM1—also described as TM1b, residues 396 to 416; TM2, residues 429 to 449; TM3, residues 478 to 498; TM4, residues 507 to 527; TM5—also known as TM5a, residues 536 to 556; and TM6, residues 631 to 651) (Figure 3) packed into a central pore, which contains the substrate binding site and constitutes its translocation pathway [9,53]. Other conserved helices partially inserted into the lipid bilayer are also considered part of the TMD: (a) TM1a, most commonly known as the elbow helix (residues F373 to N391), located in the inner membrane leaflet between the NBD and TM1b; and (b) TM5b and TM5c, connecting TM5a and TM6 at the outer membrane leaflet and forming the re-entry helix (residues T560 to G588) (Figure 1). Although this second structure is highly conserved, it is only present in the ABCG subfamily, providing a structural cap or roof at the extracellular end of the TMD [9,38,50,53].

The elbow helix is an amphipathic α-helical segment oriented parallel to the inner leaflet of the plasma membrane, serving both as an anchoring point for ABCG2 and as a structural linker between the NBD and the TMD of each monomer. Due to its amphipathic character, it is partially embedded in the lipid bilayer, with its hydrophobic face interacting with the lipid membrane and its hydrophilic face exposed to the cytosol, where it interfaces with the hot spot helix at residues R137 and E138 (Figure 1). This structural arrangement enables ABCG2 to be stably anchored to the membrane while maintaining the conformational flexibility necessary for the rotational movements associated with the transport cycle [57,61].

The re-entry helix engages all extracellular domains, suggesting a critical role in stabilizing the extracellular polar roof by restricting its conformational flexibility. This stabilization is achieved through the formation of a salt bridge between the conserved residue E585 of the re-entry helix and the R426 residue of the extracellular loop 1 (EL1)—discussed below [62] (Figure 1). The functional relevance of the re-entry helix is underscored by the fact that any mutational alteration of the highly conserved residue E585 completely abolishes the expression of mature ABCG2 at the cell surface and impairs its efflux activity [47].

In the ABCG2 protein, the TMs are interconnected by ILs and ELs (Figure 1 and Figure 3), acting as structural linkers within the polypeptide chain. Remarkably, all ILs within the ABCG family are relatively short compared to those of other ABC exporters, revealing a more compact architecture that resembles bacterial ABC importers [63]. This structural feature positions the NBDs closer to the inner leaflet of the membrane, appearing to favor efficient signal transduction between cytoplasmic and transmembrane domains [38,54,64].

The most relevant function is attributed to IL1 (residues E451 to R465), a V-shaped α-coupling helix that links TM2 and TM3 (Figure 1 and Figure 3), forming a structurally crucial element that projects from the inner membrane toward the NBD dimer [56,57,65]. This contrasts with other eukaryotic ABC transporter folds, such as those of the ABCB, ABCC, and ABCD subfamilies, where the coupling helix is typically located in the last IL [14]. Interestingly, IL1 features a flexible U-turn loop at its midpoint, precisely at the position where it interacts with the NBD surface. This configuration confers the structural flexibility required to accommodate conformational transitions, allowing IL1 to function as a molecular spring that tightly couples the NBD and the TMD. The U-turn loop harbors three highly conserved residues across all ABCG transporters—G462, Y463, and Y464—with Y464 being the most conserved among mammalian ABCG proteins [47]. G462 introduces a critical bend at the base of IL1, a structural element essential for proper folding and NBD interaction [66]. Furthermore, Y464 has been shown to be critical for ATPase activity, highlighting its key role in the catalytic cycle.

The extracellular region of ABCG2 comprises three loops: EL1, connecting TM1b and TM2 (residues 417 to 428); EL2, between TM3 and TM4 (residues 499 to 506); and the largest EL3, connecting TM5c and TM6 (residues 589 to 630), highly contributing to form a lid-like roof structure at the cell surface (Figure 1 and Figure 3) [62]. Given its greatest length and functional relevance, we focus here on EL3. Despite its low sequence conservation among ABCG subfamily members [38], EL3 contains three conserved cysteine residues—C592, C603, and C608—that form both intramolecular (C592–C608 and C592′–C608′) and intermolecular (C603–C603′) disulfide bonds (Figure 1). The intramolecular bridges are essential for loop stabilization and the formation of the upper cavity (as discussed below), which is consistent with earlier findings indicating that C592 and C608 are crucial for ABCG2 maturation and activity [67]. In contrast, the intermolecular disulfide bond appears to be non-essential for transporter function [68,69]. EL3 also includes a key N-glycosylation site at residue N596 (Figure 1), required for the proper maturation of the ABCG2 transporter [70]. Indeed, mutations at this site result in protein misfolding, functional loss, and subsequent degradation.

Several studies have demonstrated that each putative EL in ABCG2 harbors at least one charged residue that is essential for proper ABCG2 folding. Notably, conservative substitutions preserving the charge often maintain correct protein maturation, underscoring the functional relevance of electrostatic interactions within these regions. Among the most conserved residues are D419 and R426 in EL1, as well as E611, E612, and D620 in EL3. In particular, R426 stands out due to its spatial proximity to E585 in the re-entry helix, where it is proposed to form a stabilizing salt bridge. This interaction likely contributes to the structural integrity of the extracellular polar roof by restricting its conformational flexibility and enhancing overall transporter stability [62].

### 2.3. Transmission Interface

The transmission interface of ABCG2 has been extensively characterized by the group of Karl Kuchler [43,47,57]. In the ABCG2 transporter, crosstalk and mechanical coupling between the NBDs and TMDs are mediated by this interface, an essential domain in supporting and transmiting the conformational transitions preceding substrate extrusion, including the NBD lifting and rotational movements from the NBD and TMD. The unique architecture of the transmission interface of ABCG2 includes the THB formed by three highly conserved helices, (a) the hot spot helix from the NBD, (b) the elbow helix, and (c) IL1 (Figure 1), whose structural features have been described in detail above. Interestingly, key residues of the THB as well as its folding are highly conserved in the ABCG subfamily, strongly suggesting that its function is mainly related to the mechanical coupling rather than controlling substrate specificity.

The transmission interface is stabilized by two salt bridges formed between two conserved residue pairs: one between R383 in the elbow helix and E458 in IL1, and another between the glutamic acid residue E451—marking the cytoplasmic start of IL1—and K473 on the cytoplasmic side of TM3 (Figure 1). These interactions effectively anchor ABCG2 to the cytoplasmic side of the inner membrane, reinforcing the structural integrity of the transmission interface [57].

### 2.4. Structural Characterization of ABCG2 Homodimer

Structural studies have demonstrated that, upon dimerization, the two TMDs are predominantly externally lined with uncharged residues, facilitating the proper embedding of ABCG2 into the lipid bilayer [57]. However, the inner translocation channel is mainly dominated by hydrophobic residues.

Multiple studies have shown that the homodimerization of the two ABCG2-encoding polypeptide chains results in the formation of two flexible cavities: (a) the central cavity, initially described as cavity 1 by Taylor et al. [38], which is accessible from the cytosol and plays a critical role in substrate and inhibitor recognition, and (b) the upper cavity, also referred to as cavity 2, which is limited by the polar extracellular roof and primarily involved in regulating substrate release toward the extracellular space [18,20,38,45,54]. Both cavities are separated by the valve gate, which will be described in the following section.

#### 2.4.1. Central Cavity

**Figure 4:**
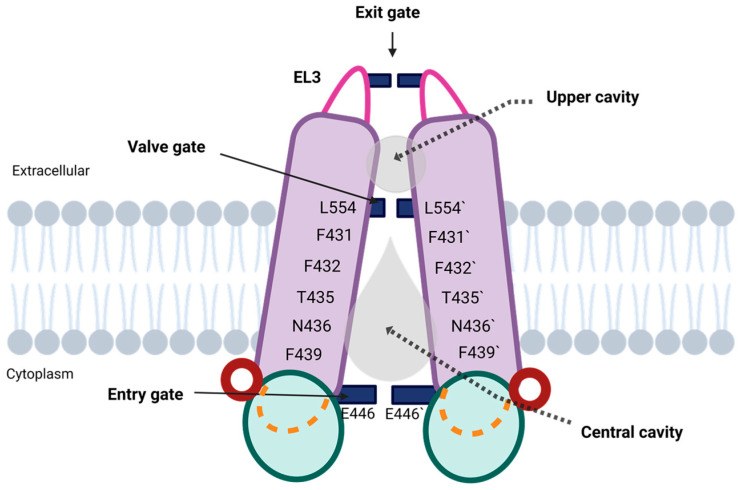
**Structural features of the ABCG2 transporter highlighting cavities and gating elements.** Central and upper cavities within the structure, along with key residues involved in the transport function, are indicated. Three gating regions—entry, valve, and exit—are shown in blue. The transporter is depicted with key structural regions color-coded for clarity: NBDs (green), elbow helix (red), IL1 (orange), TMDs (purple), and EL3 (pink). Based on Taylor et al. [38], Khunweeraphong et al. [62], and Khunweeraphong and Kuchler [43], and created in https://BioRender.com.

The architecture of the central cavity comprises the NBD dimer, the IL1, the elbow helix, and the residues facing the cavity from TM1, TM2 and TM5a. This larger cavity—also described as drug binding cavity [71]—has a teardrop-shape geometry and extends more than halfway across the membrane, reaching residues L554 and L554′ from opposing ABCG2 monomers (Figure 4) [38]. When the transporter is open, the cavity is directly accessible from the cytoplasm and functions as the primary substrate-binding site [66], being able to accommodate a wide spectrum of substrates as well as several inhibitors [7,13]. This polyspecificity is largely attributed to the abundance of hydrophobic residues lining the cavity, which provide a broad, adaptable surface for ligand interaction. Indeed, several studies have demonstrated that substrates engage in hydrophobic interactions and hydrogen bonding with approximately 10–20 residues along the translocation pathway, highlighting the cooperative contribution of multiple residues to ligand recognition, binding, and transport [18,38,43,45,54,58,72].

Despite some inconsistencies, the majority of studies indicate that the central cavity was limited by side chains from residues in three TM, including TM1 (Q398, V401, T402, and L405), TM2 (F431, F432, T435, N436, Q437, F439, S440, and V442), and TM5a (T538, L539, T542, I543, V546, and M549), respectively. Among them, almost all substrates shared ten common interacting residues in the central cavity, provided by TM2 (F432, T435, N436, F439, S440, and V442) and TM5a (T542, I543, V546, and M549) [43]. Notably, many of these residues were already described by Taylor et al. [38], in the first cryo-EM structure of ABCG2, and have been shown to be either fully conserved or replaced by similarly hydrophobic residues in other G-subfamily ABC transporters—with the exception of V546.

Among the substrate-interacting residues described, a few play clearly defined roles in substrate recognition and transport, including the three highly conserved phenylalanine residues in TM2—F431, F432, and F439, T435, and N436 (Figure 4). In particular, the centrally positioned F439 pair is critical for the initial binding and stabilization of ligands through π–π stacking interactions [73]. This interaction, which is imperative for all ligands, may explain the structural preference of ABCG2 for flat, polycyclic, and hydrophobic substrates [45]. Thus, the F439 pair is proposed to have a valve-like function, preventing backwards movements of the substrate [18]. Additionally, the F432 pair supports unidirectional transport toward the upper cavity by maintaining hydrophobic interactions with the substrates. Although F431 does not appear to interact directly with substrates, it is essential for transport function, likely contributing to the structural stability of the leucine valve through side-chain packing. Intriguingly, F431 has also been implicated in inhibitor binding, as mutations at this position reduce susceptibility to known ABCG2 inhibitors [74,75].

Finally, docking analyses have identified T435 and N436, located just above F439, as potential substrate-selecting residues, undoubtedly contributing to the specificity and selectivity of the ABCG2 transporter [43].

#### 2.4.2. Upper Cavity

The smaller upper cavity, located near the extracellular side, extends from the di-leucine valve to the polar extracellular roof (Figure 4) [62]. In the inactive conformation of the ABCG2 transporter, the upper cavity is sealed off from both the cytoplasmic and extracellular sides. This fact, together with its less pronounced hydrophobic surface compared to the central cavity, which contributes to a lower substrate affinity, suggests a key role in facilitating substrate release into the extracellular space [38].

Interestingly, the upper cavity had not been clearly described in other ABCG transporters, likely due to structural differences in EL3, a key element in forming the extracellular roof. This may explain why, despite the availability of numerous ABCG2 structures with bound substrates and inhibitors in the central cavity, the upper cavity remained largely uncharacterized until 2022, when Dudas et al. used molecular dynamics simulations to model substrate binding within this region. This group realized that the substrate behavior within the upper cavity markedly differs from that observed in the central cavity. While the substrates are tightly bound and closely surrounded by specific aminoacidic residues in the central cavity, they appear more loosely bound in the upper cavity, dynamically interacting with boundary residues such as S420, C592, Y605, A606 (from both monomers), and K616 (from one monomer) [18].

#### 2.4.3. Regulatory Gates

Structurally, the central and upper cavities are limited by three different regulatory gates:

1. The “entry” gate

In recent years, multiple studies have proposed that, prior to entering the central cavity, ABCG2 substrates interact with different binding sites on the transporter, this first interaction being a crucial step for membrane translocation. These binding pockets have been variously referred to as the “access site” by Kapoor et al. [50], as “site 1” and “site 2” by Nagy et al. [72], and as the “entry gate” by Khunweeraphong and Kuchler [43]. According to Nagy et al., site 1 is a cytoplasmic region and site 2 corresponds to a pocket formed by residues in TM1b, TM2, and TM3, surrounding the essential residue R482, whose mutation has been shown to broaden substrate specificity [72]. Kapoor et al. also propose that access to the central cavity is controlled by the helical interface between the intracellular ends of helices TM2 and TM3 [50]. However, Khunweeraphong places the “entry” gate at the inner leaflet of the membrane, near the highly conserved transmission interface THB, in agreement with the findings reported by other groups [43,76]. It is well-known that the THB mediates numerous hydrophobic interactions with substrates, involving residues in the NBD (V129, V130, M131, T133, Q181, and F182), the elbow helix (L388, N389, N391, and P392), and IL1 (Q393, A394, S395, E446, V450, E451, and D477), along with polar contacts from D128, T180, and N387. Among these, the glutamate at E446 (Figure 4) in IL1 has been recently proposed as a potential binding region to control substrate access at the “entry gate”, as supported by mutational analyses [77]. Interestingly, Dutra et al. reported that, in the case of non-competitive inhibition, residue E446 plays an important role in the stabilization of inhibitors inside the central cavity [71]. Other negatively charged residues in this region—E451 and D477—have been shown to be essential [43].

2. The “valve” gate

The central and upper cavities are separated by a valve-like structure, known as the valve gate, formed by the conserved residues from G553 to T559 and supported, as previously indicated, by the F431 residue, which lies immediately below the L555 residue. This second gate acts as a hydrophobic seal, maintaining a strict separation from the central cavity and controlling substrate access to the upper cavity [62]. The key feature of the valve gate is a di-leucine motif—also known as the leucine plug—comprising residues L554 and L555 from opposite monomers, which are positioned in close proximity within the translocation channel (Figure 4). Interestingly, some studies suggest that L554 is not essential for ABCG2 biogenesis or function, while the presence of a hydrophobic residue at position 555 is critical for proper folding and substrate translocation to the upper cavity. Moreover, L554 is replaced by phenylalanine in the vast majority of other ABCG transporters (136 out of 141 sequences) [47].

3. The “exit” gate

The exit gate is a lid-like structure constituted by the extracellular polar roof, which plays a critical role in modulating the shape and size of the upper cavity and in controlling substrate release. As discussed in the previous section, this polar roof comprises EL1, EL2, and EL3, along with the re-entry helix (Figure 1). Structural analyses have identified several common interacting residues across multiple substrates within this roof region, including residues from EL1 (S420, T421, I423, Q424, and A427) and EL3 (Y605, A606, T607, Y613, K616, and Q617) [18,43]. Interestingly, recent studies suggest that, prior to substrate release, all interactions between substrates and EL3 residues disappear. This implies that the exit gate operates through dynamic conformational rearrangements of EL3, which expand the upper cavity and reduce substrate affinity, thereby facilitating substrate extrusion through the transiently open roof.

## 3. Transport Mechanism

### 3.1. Transport Model Proposal

Understanding the structure is key to elucidating the transport mechanisms, as evidenced by structural studies of ABCG2 that have, to date, enabled a deeper insight into its specific mode of transport. The initial and broadly applicable model of substrate translocation for ABC transporters is the “alternating access model”. However, the idea of a universal transport mechanism for all of them is increasingly being questioned, and distinct molecular mechanisms and transport cycles are likely to exist [57,76]. In the case of ABCG2, the historical absence of a reliable structural model has led to the formulation of multiple hypotheses regarding its transport mechanism; among these, the alternating access model has emerged as the most widely accepted explanation [47]. Building upon this model, more recent research has progressively incorporated additional details and refined our understanding of the process.

**Figure 5:**
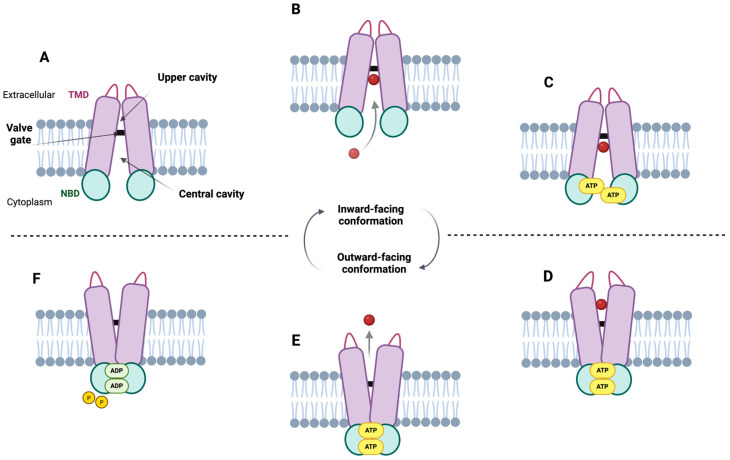
**Overview of the ABCG2 substrate translocation process.** (**A**) In the inward-facing conformation, the nucleotide-binding domains (NBDs, green) are separated and oriented toward the cytoplasm, exposing the central cavity to the cytoplasm while the upper cavity remains closed. (**B**) Substrates (red) enter from the cytoplasm and bind near the leucine plug (represented by the black bar). (**C**) Upon binding two ATP molecules to the NBDs, their dimerization induces a conformational shift from inward- to outward-facing conformation. (**D**) This rearrangement, due to the coupling between the NBDs and transmembrane domains (TMDs, purple), closes the central cavity and opens the upper cavity, allowing substrate translocation and (**E**) release into the extracellular space. (**F**) Finally, ATP hydrolysis resets the transporter to its original inward-facing state [38,54,62]. Created in https://BioRender.com.

Within this framework, it has been established that the ABCG2 transporter can adopt two conformational states: an inward-facing conformation (IF), also known as the inward-open state, or drug-trapped or ATP-free state; and an outward-facing conformation (OF), referred to as the outward-closed state. In the IF conformation, the central cavity opens toward the cytoplasm, while the upper cavity remains inaccessible due to the valve gate. In this state, NBDs are separated and oriented toward the cytoplasm, favoring the entry of the substrate into the central cavity, which is completely closed off from the extracellular environment (Figure 5A,B). In contrast, in the OF conformation, NBDs are tightly associated on the cytoplasmic side, preventing the backflow of the substrate that has entered the central cavity (Figure 5D). Meanwhile, the upper cavity becomes accessible and opens toward the extracellular space, facilitating substrate release (Figure 5E) [38,57].

Essentially, in the absence of a ligand, ABCG2 adopts an IF conformation, leaving the central cavity open on the intracellular side of the membrane (Figure 5A). However, in the presence of a substrate (Figure 5B), a series of conformational changes are triggered toward OF configuration. ATP binding plays a crucial role in this process, as it promotes the dimerization of the NBDs, leading to the closure of the NBD interface (Figure 5C). This structural rearrangement converts the IF conformation into an OF state (Figure 5D), thereby facilitating substrate release to the extracellular space (Figure 5E) [38,62]. The proposed mechanism involves the entry of the substrate from the cytoplasm into the central cavity, where it becomes trapped in the IF conformation (Figure 5B) [9]. Subsequently, the binding of two ATP molecules to the NBDs promotes their dimerization and initiates an upward shift of IL1, which, together with the elbow helix, contributes to triggering the conformational transition. Therefore, when drugs are recognized or retained within the central cavity, a process in which residue E446 (Figure 4) appears to play a key role, the interfaces (Figure 1) formed by IL1, the elbow helix, and the NBDs become essential for the conformational switch [57]. Due to the connection between the NBD and the TMD, the rotation of the NBD is transmitted to the TMD, leading to the closure of the central cavity and the opening of the upper cavity, thereby driving the transition to the OF conformation. This conformational shift results in the closure of the intracellular side of the transporter, allowing the substrate to move from the central cavity to upper cavity (Figure 5D). Once the substrate clears the leucine plug area and enters the upper cavity, the valve gate closes, and the substrate is released (Figure 5E) into the extracellular space through a hydrophobic mismatch [9,38,54,57]. Finally, ATP hydrolysis occurs after the release of the substrate, providing the energy required to restore the IF conformation of the transporter (Figure 5F). However, this step remains a subject of debate among some authors, and it will be addressed in the following sections.

### 3.2. Updating the ABCG2 Transport Mechanism

Taking into account the proposed general mechanism for ABCG2, several complementary studies have been conducted, offering a more precise understanding of the conformation and spatial arrangement of ABCG2, including its interaction sites with substrates and inhibitors, as well as its transport mechanism. Therefore, Manolaridis et al. [54] demonstrated that substrate coordination is primarily mediated by the side chains of residues N436 and F439 (Figure 4), and the binding of the substrate was observed to enhance ATP hydrolysis, thereby providing evidence that substrate binding occurs before ATP binding. Furthermore, regarding substrate binding, Kapoor et al. [50] slightly differ from the general model, suggesting that the substrate can bind to three distinct sites during the transport process: the access site (located between TM2, TM3, and TM6), the binding site (corresponding to central cavity), and the extrusion site (corresponding to upper cavity). These authors suggest that the substrate initially interacts with the transporter at the access site—where residue R482 is situated—rather than within the central cavity [50]. Supporting this, mutagenesis studies have shown that R482 plays a key role in determining the substrate selectivity of ABCG2 [78]. Then, substrate binding to the access site promotes ATP binding, which provides the power for the translocation of the substrate drug from the access site to the central cavity. Accordingly, the proposed models indicate that the substrate may interact with multiple distinct sites within ABCG2 throughout the transport cycle [50].

Moreover, Khunweeraphong et al. [62] describe the importance of substrate translocation as involving two critical steps associated with two significant barriers. Thus, the first is the conserved valve gate including the di-leucine motif. As previously mentioned, this valve creates a hydrophobic seal at the top of the central cavity, preventing flow between the two cavities in the IF conformation. In contrast, the conformational change to the OF state weakens this hydrophobic seal, allowing peristaltic pressure to push the substrates into the upper cavity. The second barrier is the flexible polar roof, whose opening —promoted by peristaltic pressure—facilitates the release of the substrate into the extracellular space. Notably, in the IF conformation, the upper cavity is nearly completely closed due to the presence of the di-leucine valve, perhaps to prevent the re-entering of substrates into the translocation pore, which is necessary in order to ensure unidirectional transport [62].

While most studies on multidrug transporters have focused on strategies such as locking intermediate states or limiting conformational flexibility, ABCG2 structures obtained under turnover conditions allow the observation of the protein while it is functionally active, that is, in the presence of ATP and substrate. Some studies describe two distinct IF conformations, each one containing both a bound transport substrate and two ATP molecules: turnover-1 (TO1) and turnover-2 (TO2). TO1 exhibits a greater separation of the NBDs and a wider substrate-binding cavity compared to TO2. They have been seen as transitional stages in the transport cycle, with TO1 forming initially, followed by TO2, as the protein moves toward the OF state [20,79]. Thus, for endogenous substrates like estrone-3-sulfate (E1S), it was shown that TO2 appears to immediately precede the rate-limiting step of the transport cycle. In contrast, for exogenous compounds such as topotecan, TO1 was the dominant conformation, and the transition to TO2 may serve as a checkpoint to assess whether these compounds are suitable for transport. Therefore, compounds that bind to the central cavity and permit the adoption of the TO2 conformation may be transported, whereas strongly bound inhibitors prevent this transition and, thereby, block transport [20]. In both studies, the importance of the phenyl rings of the two opposing F439 residues from the ABCG2 monomers (Figure 4) was proposed to help bind the substrates, as previously observed [18,73]. They also showed that both tested substrates, topotecan and tariquidar, change their orientation between the TO1 and TO2 conformations, a rearrangement that may be required to fit into the narrower substrate-binding cavity and allow further translocation [20,79].

Finally, a recent study on the structure of ABCG2 has proposed a new substrate translocation model, characterized by three distinct phases, clamping, pushing, and sealing, which are associated with the presence of a gating mechanism [43]. The study outlines a pathway that includes two main cavities, the central and the upper cavities, separated by three distinct regulatory gates, as indicated before: the entry gate, the valve gate, and the exit gate. This study suggests that drug efflux requires the formation of a “clamp–push–seal” aromatic arm through three phenylalanine pairs—F431, F432 and F439—along with the selector residues T435 and N436 (Figure 4). In the IF state, an entry gate allows ligand access to the central cavity, which provides a spacious binding pocket for substrates and inhibitors. The aromatic side chain of the F439 pair forms a “clamping arm” to trap ligands through stacking interactions in the central cavity, preceding specific interactions with T435 and N436 that help classify substrates and inhibitors. ATP binding induces NBD dimerization, which closes the entry gate and forms a substrate-trapped closed pore. As a result, the aromatic side chains of F439 moved, compressing the central cavity, generating peristaltic pressure and facilitating ligand transfer to the F432 pair. This pair establishes hydrophobic interactions with drugs during the IF-to-OF switch and exhibited a long-range upward movement which constitutes a ‘‘pushing action’’ that triggers unidirectional transport from the central cavity to the upper cavity. Finally, F431 residue contributes to the valve structure by using its side chain as a hydrophobic seal for the valve gate. After crossing the valve gate and expanding the upper cavity, the exit gate opens, enabling the release of substrates and completing the transport cycle.

### 3.3. ATP Hydrolysis in ABCG2: A Controversial Step

As previously mentioned, the NBD domain is responsible for ATP binding and hydrolysis and is composed of several conserved motifs [51,52]. However, despite recent advances in structural studies, it remains controversial whether ATP binding or hydrolysis is the driving force behind the conformational changes that enable substrate transport across the membrane, or whether ATP hydrolysis serves to reset the transport cycle.

On one hand, several studies suggest that ATP binding initiates the conformational change, as it promotes NBD dimerization, leading to a conformational shift of the transporter from the IF to the OF state [45,54,64]. Therefore, ATP binding may be providing the energy for the substrate extrusion step [9]. Likewise, several authors support the notion that ATP hydrolysis occurs after substrate release, as it is necessary to return the ABCG2 transporter to its IF conformation [18,20,38,54,62,80]. On the other hand, in the model proposed by Kapoor et al. [50], ATP hydrolysis would cause conformational change to facilitate the movement of the substrate from the central cavity to the upper cavity. Consequently, the IF state of the transporter is then restored by the release of ADP.

At last, even other studies suggest that ATP binding and hydrolysis may be independent events, without a direct relationship to substrate binding [57]. Furthermore, given the high intracellular concentrations of ATP available in the cell, it cannot be ruled out that ATP molecules are constitutively bound to the NBDs of the transporter, even in the absence of the substrate in the central cavity [20]. Thus, ATP hydrolysis cannot be definitively considered a requirement for substrate translocation, as it has been shown that ABCG2 is capable of constitutively hydrolyzing ATP without performing transport activity [47].

In conclusion, although the exact sequence of these events remains under active investigation, it is generally accepted that the conformational change is triggered upon substrate entrapment within the central cavity. ATP binding and the subsequent hydrolysis are believed to mediate conformational change, thereby driving the transport cycle and promoting the extrusion of a wide range of substrates from the cell.

## 4. Structural Interactions for Substrates and Inhibitors

Given the broad substrate spectrum of ABCG2, a key unresolved question is which molecular features determine whether a compound acts as a substrate or an inhibitor. Structural studies have identified distinctions in the binding mechanisms of these two classes of ligands. While most models propose that both substrates and inhibitors interact with the transporter within the upper cavity—underscoring its dual role in multidrug recognition [54]—the principal difference appears to lie in the extent to which each molecule occupies the binding site.

In the case of substrates, their smaller size, reduced interaction surface, and deeper binding position within the upper cavity enable efficient translocation during the transport cycle [54]. Unlike inhibitors, substrates are not tightly immobilized in the binding pockets, appearing to be shifting in the central cavity [58]. This supports a dynamic binding model, in which residues lining the central cavity undergo conformational rearrangements upon transition to the OF state [18,76]. Such changes are thought to decrease substrate affinity, favoring a progression toward the upper cavity. This dynamic model also helps explain the diversity of binding sites observed among structurally distinct substrates, which probably stems from differences in size and chemical composition. In particular, it has been shown that larger exogenous compounds interact with more spatially distant residues along the translocation pathway [58].

Nevertheless, inhibitors adopt an extended conformation that acts as a wedge, preventing ATP-induced NBD dimerization and locking the transporter in the IF conformation [54]. Structural models further support this hypothesis by showing that, in the IF conformation, inhibitors bind within the substrate-binding central cavity, as demonstrated for MZ29—a derivative of the selective ABCG2 inhibitor Ko143—thereby blocking access for substrates to its translocation pathway by locking the IF state of the TMDs, which prevents the closure of the NBDs [45]. Thus, inhibitors prevent ATP binding/hydrolysis and further conformational change to the OF state [64]. An alternative interpretation is provided by the model proposed by Kapoor et al. [50], which suggests that only substrates are capable of entering the central cavity through the access site, a process essential for the opening of the leucine gate. In contrast, inhibitors are thought to bind directly to the binding site, thereby blocking ATP hydrolysis and preventing the transport of other substrates.

Overall, the mechanisms underlying the remarkably broad substrate selectivity of ABCG2 remain elusive; however, the potential existence of a multi-ligand binding site within the central cavity has been proposed as a hypothesis to explain the broad range of molecules that interact with the transporter [43,45], although more studies focused on substrate-bound structures are still needed.

## 5. Perspective

Therefore, what lies ahead for ABCG2 research? Despite the significant progress in recent years, particularly in structural studies that have provided new insights and identified key residues involved in the transport mechanism, some aspects remain to be elucidated. A comprehensive understanding of the interactions between ABCG2 and its wide range of substrates and inhibitors continues to be elusive. Future research will be essential in order to unravel the detailed molecular mechanisms governing these interactions, thereby advancing our understanding of ABCG2′s role in drug transport and reinforcing its potential as a therapeutic target. From a forward-looking perspective, a thorough structural characterization will be indispensable not only for the identification of novel drug candidates as ABCG2 substrates, but also for the rational design of highly specific ABCG2 inhibitors with therapeutic relevance.

## Data Availability

Not applicable.

## Abbreviations

The following abbreviations are used in this manuscript:

ABC	ATP-Binding Cassette
BCRP	Breast Cancer Resistance Protein
Cryo-EM	Cryo-Electron Microscopy
E_1_S	Estrone-3-Sulfate
EL	Extracellular Loop
IL	Intracellular Loop
P-gp	P-glycoprotein
MDR	Multidrug Resistance
MRP1	Multidrug-Resistance-Associated Protein 1
NBD	Nucleotide-Binding Domain
THB	Triple Helical Bundle
TMD	Transmembrane Domain
TM	Transmembrane Helix
TO1	Turnover-1
TO2	Turnover-2
